# Pediatric acute transverse myelitis

**DOI:** 10.17712/nsj.2017.3.20170294

**Published:** 2017-07

**Authors:** Bashaer Albulushi, Brahim Tabarki

**Affiliations:** *From the Division of Neurology, Department of Pediatrics, Prince Sultan Military Medical City, Riyadh, Kingdom of Saudi Arabia*

## Case Presentation

A 12-year-old boy, previously healthy, developed fever and Upper Respiratory Tract Infection symptoms followed by bilateral lower limb weakness, with numbness and right upper limb weakness. He also developed urinary retention. On examination, the reflexes in both lower limbs couldn’t be elicited and there was sensory level up to midthoracic area. His Glasgow coma scale was 15/15. The cerebrospinal fluid analysis showed pleocytosis and high protein. MRI spine is shown below.

**Figure 1:**
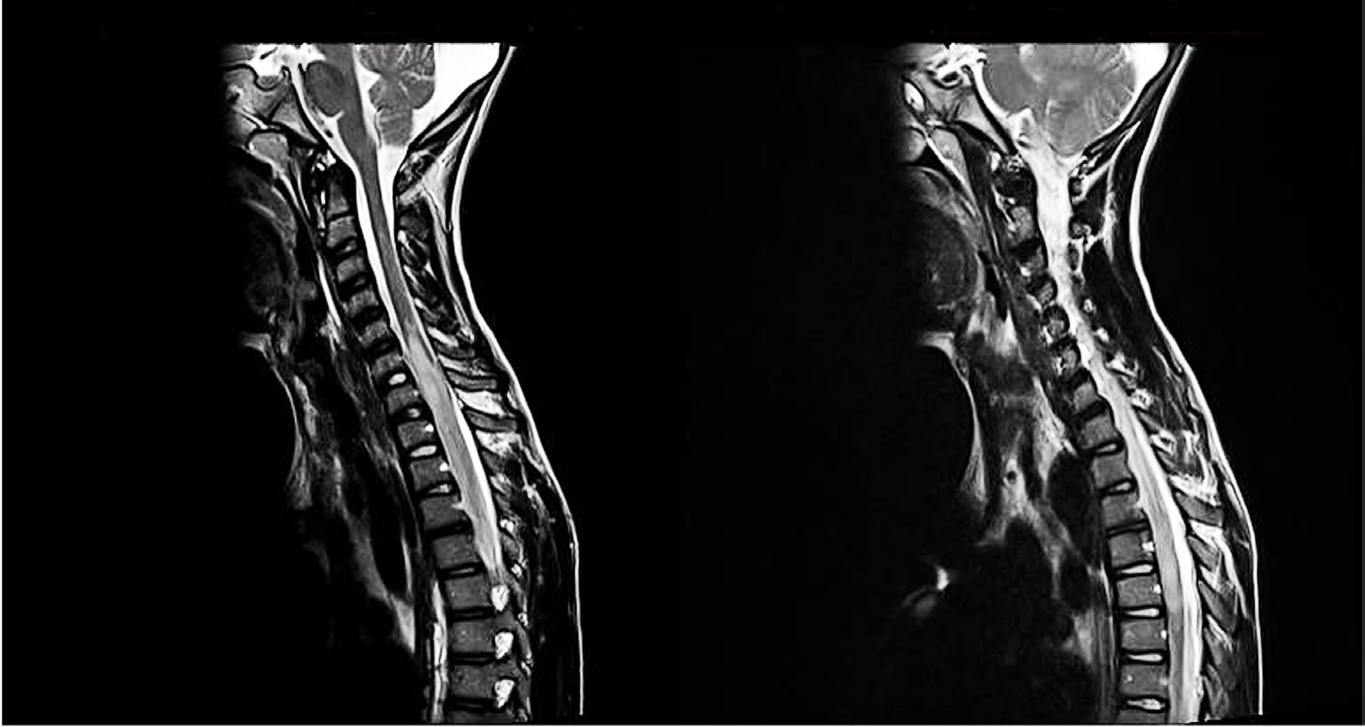


## Questions

1. Which of the following is the most probable diagnosis?
  - Guillain-Barré syndrome
  - Spinal cord arteriovenous malformation
  - Acute transverse myelitis
  - Poliomyelitis
2. Which of the following is the most commonly affected site of the spinal cord?
  - Cervical
  - Lumbar
  - Thoracic
  - Cervical and cervicothoracic
3. Which of the following is first line treatment?
  - High dose of steroids
  - Intravenous acyclovir
  - Intravenous immune globulin
  - Intravenous antibiotics
4. Following therapy and during recovery, which of the following is the first to recover?
  - Sensory impairment
  - Pain
  - Bladder function
  - Motor function
5. In patients diagnosed with acute transverse myelitis, what’s the risk to develop neuromyelitis optica?
  - 30%
  - 55%
  - 10%
  - 3%

## Answers & Discussion

1. **c**
  Acute transverse myelitis is an immune-mediated central nervous system disorder classically described as demyelinating. It can present with back pain as the first symptom followed by motor and sensory deficit or bladder/bowel dysfunction. Sensory symptomatology can be either positive (burning, paresthesia, hyperesthesia, allodynia) or negative (numbness).^1^,^2^
2. **d**
  Cervical and cervicothoracic lesions represent the majority of acute transverse myelitis lesions (64%-76%).^1^,^2^
3. **a**
  The standard empiric therapy for acute transverse myelitis is high dose corticosteroids. Pediatric patients are usually treated with a 30 mg/Kg/dose (maximum 1000 mg) of methylprednisolone intravenously once a day for 3 to 5 days.^1^-^3^
4. **b**
  Following immunotherapy, pain is the first symptom to resolve, followed by an improvement in motor deficits. Bladder function and sensory deficits may take longest to improve.^1^-^3^
5. **d**
  The largest cohort study (French & UK collaboration) describing 95 pediatric patients with acute transverse myelitis found that 14% relapsed with multiple sclerosis and 3% with neuromyelitis optica.^3^
